# Intraflagellar transport speed is sensitive to genetic and mechanical perturbations to flagellar beating

**DOI:** 10.1083/jcb.202401154

**Published:** 2024-06-03

**Authors:** Sophie Gray, Cecile Fort, Richard John Wheeler

**Affiliations:** 1 Nuffield Department of Medicine, Peter Medawar Building for Pathogen Research, University of Oxford, Oxford, UK

## Abstract

Two sets of motor proteins underpin motile cilia/flagella function. The axoneme-associated inner and outer dynein arms drive sliding of adjacent axoneme microtubule doublets to periodically bend the flagellum for beating, while intraflagellar transport (IFT) kinesins and dyneins carry IFT trains bidirectionally along the axoneme. Despite assembling motile cilia and flagella, IFT train speeds have only previously been quantified in immobilized flagella—mechanical immobilization or genetic paralysis. This has limited investigation of the interaction between IFT and flagellar beating. Here, in uniflagellate *Leishmania* parasites, we use high-frequency, dual-color fluorescence microscopy to visualize IFT train movement in beating flagella. We discovered that adhesion of flagella to a microscope slide is detrimental, reducing IFT train speed and increasing train stalling. In flagella free to move, IFT train speed is not strongly dependent on flagella beat type; however, permanent disruption of flagella beating by deletion of genes necessary for formation or regulation of beating showed an inverse correlation of beat frequency and IFT train speed.

## Teaser

We used high-frame-rate, dual-color fluorescence microscopy to visualize IFT trafficking in the beating flagella of *Leishmania mexicana*, a uniflagellate human parasite. This revealed differing sensitivity of IFT train speed to genetically induced, mechanical, and natural changes in beating.

## Introduction

Motile eukaryotic flagella and cilia have a complex architecture. They are assembled, maintained, and function through the action of two types of motor proteins. Intraflagellar transport (IFT) protein complexes called trains (Rosenbaum and Witman, 2002) travel bidirectionally along the flagellum powered by kinesin-II (anterograde) (Kozminski et al., 1993) and dynein (retrograde) (Pazour et al., 1998), carrying cargo to the tip then returning IFT machinery to the base (Cole et al., 1998). Flagellar beating is driven by dynein complexes that slide flagellar axoneme microtubule doublets relative to their neighbor (Lindemann, 2007; Smith and Yang, 2004). These movements occur on different time scales; IFT trains typically take several seconds to travel the length of the flagellum (Buisson et al., 2013) while one flagella beat cycle typically takes tens of milliseconds (Friedrich and Riedel-Kruse, 2014). Despite the different time scales, both arise from comparable motor protein travel speeds along microtubules (Webb et al., 2020). However, IFT involves processive movement while beating occurs by rapid activation/inactivation cycles (Prevo et al., 2017).

Visualizing IFT within a beating flagellum is extremely challenging. First observed in paralyzed flagella of *Chlamydomonas reinhardtii* by light microscopy (Kozminski et al., 1993), IFT trains were later correlated with electron-dense complexes found by electron microscopy (EM) (Kozminski et al., 1995). While molecular cell biology analysis of IFT machinery has advanced dramatically, approaches for visualizing IFT movement have not. It is still essentially exclusively carried out in naturally immotile cilia/flagella, paralyzed flagella mutants, or mechanically immobilized flagella (often adhered to a slide or coverslip).

We are not aware of any quantitative analysis of IFT in a cilium/flagellum in its physiological state of beating. Therefore, the true nature of classic IFT features—train speed, processivity, frequency, and stalling—remain unknown for motile flagella undergoing their normal behavior.

*Leishmania mexicana* is an excellent species for IFT analysis. As members of the *Trypanosomatidae* unicellular parasite family, they possess a single flagellum necessary for motility, morphogenesis, and surface attachment (Barrett and Croft, 2012; Landfear, 2022; Stevens, 2008; Sunter et al., 2018). *L. mexicana* is of medical importance as one of the etiological agents of leishmaniasis, a neglected tropical disease. Importantly, the *Leishmania* procyclic promastigote flagellum is motile with a canonical nine outer microtubule doublet pairs and a central pair (9 + 2) axoneme structure (Simpson et al., 1982). They are readily grown in culture, can be mechanically immobilized by adherence to glass, and are highly genetically tractable (Beneke et al., 2017), allowing facile deletion of genes necessary for flagellar beating (Beneke et al., 2019). Furthermore, *Leishmania* and related species like *Crithidia* undergo two different beat types: high frequency (20–25 Hz) symmetric tip-to-base for swimming and low frequency (5 Hz) asymmetric base-to-tip for reorientation (Gadelha et al., 2007; Holwill and McGregor, 1975).

The motor proteins for both IFT and beating interact with the axoneme outer doublets (to drive walking and sliding, respectively), use ATP as an energy source, and occupy the same cellular environment. Therefore, one may suspect spatial, energetic, or cross-regulatory interaction between them.

Spatial interaction is plausible as IFT trains are large complexes that must access the microtubule doublets decorated with inner dynein arms, nexin links, and radial spokes (RS) facing the central pair and outer dynein arms (ODAs) on the outer face. IFT can only access the outer face and are the same width as the microtubule doublet—thus fitting tightly between ODAs on neighboring doublets (Jordan et al., 2018; Webb et al., 2020; Lacey et al., 2023). IFT can be restricted to specific doublets. Unlike *Chlamydomonas* (Stepanek and Pigino, 2016), IFT in the *Leishmania-*related parasite *Trypanosoma brucei* is restricted (to doublets 3, 4 and 7, 8 and 9) (Bertiaux et al., 2018), potentially due to the extra-axonemal paraflagellar rod attached to doublets 5–6. Finally, the entire flagellum is membrane encapsulated. These spatial constraints may change as the axoneme bends in a beat-type-specific manner, periodically generating a different spatial environment.

Flagellar beating is energy intensive, consuming 2.3 × 10^5^ ATP per beat (in demembranated sea urchin sperm flagella) (Chen et al., 2015), and proportionally increasing with beat frequency (≈5 × 10^6^ ATP/s at 20 beats per second) (Chen et al., 2015; Takano et al., 2021; Brokaw and Simonick, 1977). IFT is likely less energy intensive. In *Chlamydomonas,* anterograde trains are ≈300 nm long (Jordan et al., 2018) with a kinesin every 6 nm (Lacey et al., 2023; Jordan et al., 2018), a speed of ≈2.5 µm/s and a frequency of 1.6 trains/s (Engel et al., 2012). Kinesin, similar to dynein, travels ≈8 nm for each hydrolyzed ATP (Coy et al., 1999; Toba et al., 2006). Predicted ATP usage by anterograde IFT is therefore ≈3 × 10^4^ per second. The retrograde IFT train structure is not yet known, but ATP usage is likely comparable. Total IFT usage is therefore ≈6 × 10^4^ ATP per second, orders of magnitude less than for beating. Cytoplasmic ATP diffusing into the flagella base (Takao and Kamimura, 2008) is likely the energy source for both processes.

Flagellum beating is regulated in response to environmental changes, often using cAMP and Ca^2+^ as intraflagellar secondary messengers (Smith, 2002). IFT can also be regulated by Ca^2+^ (Collingridge et al., 2013; Fort et al., 2021), hypothetically allowing flagellar beat and IFT coregulation. The transport capacity of IFT inversely scales with flagellum length in *Chlamydomonas* as a mechanism of length control (Engel et al., 2009). Perhaps flagellum beating interacts with IFT movement too, as a mechanism for length control?

We tested these hypotheses in *L. mexicana*. Using carefully designed cell lines, expressing fluorescent protein-tagged IFT, and high-frame-rate (100–400 Hz), dual-color epifluorescence, we visualized IFT in freely beating flagella. IFT behavior was overall similar to mechanically immobilized flagella; however, in the physiological state of beating, IFT was faster with less stalling. We tentatively detected slightly faster IFT train speeds for flagella undergoing the tip-to-base symmetric rather than base-to-tip asymmetric beat. However, permanent genetically caused changes to flagellum beat did notably alter IFT: increased IFT train speed correlated with reduced flagella beating, suggesting competition for energy. IFT train speed is therefore insensitive to short switches of flagellum beat type but is sensitive to disruption of axonemal machinery necessary for normal beating.

## Results and discussion

**Figure 1.**
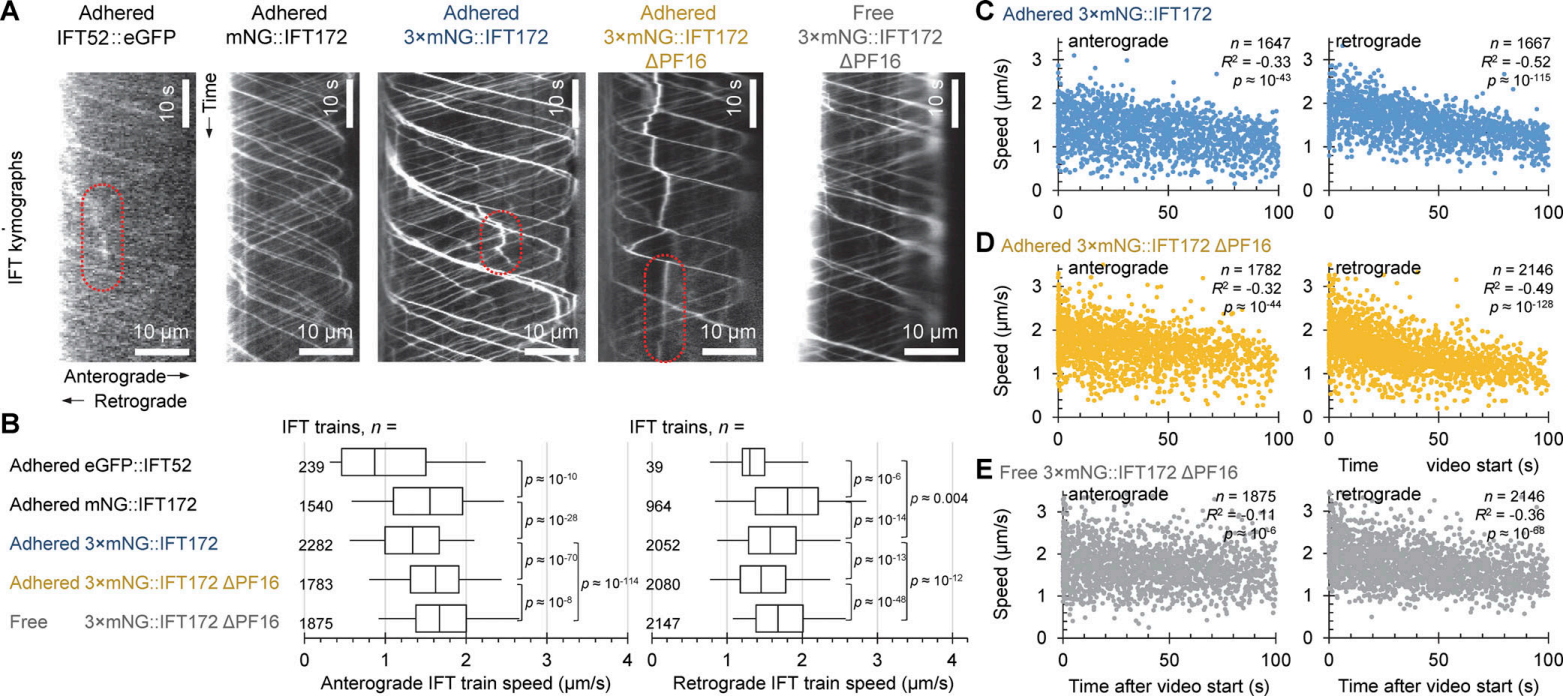
**IFT train speed depends on fluorescent tag and immobilization method in *Leishmania.* (A)** Example IFT kymographs from cell lines expressing a fluorescently tagged IFT protein, immobilized by adherence to glass, paralysis by PF16 deletion, or both. Occasionally IFT trains stall mid-flagellum; examples are outlined in red. **(B)** Box plots of anterograde and retrograde IFT train speed, measured from 102.4-s videos of glass-adhered flagella of IFT52::eGFP, mNG::IFT172, 3×mNG::IFT172, 3×mNG::IFT172 ΔPF16, and free flagella of 3×mNG::IFT172 ΔPF16. Boxes represent the lower quartile, median, and upper quartile, and whiskers mark the 5th and 95th percentile. P value of *T* tests with each of the other cell lines are displayed on the right of the graph if they were significant, i.e., < 0.05. **(C)** Scatter graphs of the speed of anterograde trains and retrograde IFT trains over time in flagella of 3×mNG::IFT172 cells with their flagella adhered to glass. Each data point represents an individual IFT train. *n* indicates the number of IFT trains, R^2^ indicates Pearsons correlation coefficient. The P values for these correlations are shown. **(D)** As for C but in 3×mNG::IFT172 ΔPF16 with flagella adhered to glass. **(E)** As for C, but in the free flagella of 3×mNG::IFT172 ΔPF16. Animated version of A in Video 1.

To visualize IFT during beating, high-video frame rates (≈200 Hz) are necessary for a short enough exposure to freeze *Leishmania* flagellum beating. Our previous *L. mexicana* IFT analysis used IFT52::eGFP and SMP1::mCh markers of IFT and the flagellar membrane, respectively (Wheeler et al., 2015). Phosphate-buffered saline (PBS)–washed cells adhere to glass slides, immobilizing the flagellum. Digital straightening of the flagella allows the generation of IFT kymographs from low-frame-rate (2.5 Hz) epifluorescence videos, showing canonical IFT (Fig. 1 A and Video 1), similar to *Chlamydomonas* (Dentler et al., 2009; Iomini et al., 2001) and *T. brucei* (Morga and Bastin, 2013).

**Video 1.** **Fluorescently tagged IFT trains moving in the flagella immobilized by adherence to glass, paralyzed by PF16 deletion, or both.** This video extends Fig. 1 showing the same example cell for each cell line. Row 1 shows the image sequence of a field of view of the example cell visualized under epi-illumination at a low frame rate (2.5 or 5 Hz); row 2 shows the fluorescent signal from the digitally straightened flagella of the example cell from each frame. In row 3, a white line traces down the IFT kymograph with time in milliseconds displayed to show how the fluorescence signal from the digitally straightened flagella is translated into the kymograph for each example cell.

However, IFT52::eGFP fluorescence is much too weak for high-frame-rate analysis. To increase IFT fluorescence signal, we used genome-wide *T. brucei* protein localizations (Billington et al., 2023) to identify N-terminal tagging of IFT172 as giving bright fluorescence. We used the bright and photostable mNeonGreen (mNG) (Shaner et al., 2013), maximizing the signal by using three copies separated by glycine-serine linkers (3×mNG::IFT172) (Paterou et al., 2023, *Preprint*). As IFT protein tagging can perturb IFT, we measured flagellum length: 14.3 ± 4.4 μm (*n* = 68, mean ± standard deviation) in parental cells, compared with 13.0 ± 3.5 μm (*n* = 61, P > 0.05, two-tailed *t* test) and 10.9 ± 4.1 μm (*n* = 53, P < 10^–5^, two-tailed *t* test) for cell lines expressing mNG::IFT172 or 3×mNG::IFT172, respectively. Kymographs from immobilized flagella showed more visible IFT trains and higher train speeds in comparison to IFT52::eGFP (Fig. 1, A and B).

### IFT trains are slower in adhered flagella than genetically paralyzed flagella

While intended for visualization of IFT trains in beating flagella, an intense and photostable 3×mNG signal allowed long-term imaging to test the effect of flagellum immobilization. IFT train speed decreased over time, affecting retrograde more than anterograde trains (Fig. 1, C–E). PBS washing and flagella adhesion therefore have a detrimental effect on IFT train speed, so we asked if genetic paralysis has a similar effect.

We deleted both alleles of PF16 (ΔPF16), a central pair protein essential for flagellar beating (Beneke et al., 2019), in the 3×mNG::IFT172 cell line. In adhered flagella, ΔPF16 had little difference in IFT train speed. For cells in a growth medium (where flagella are free), anterograde and retrograde IFT train speeds were significantly higher and decreased over time more slowly (Fig. 1, B and E). Therefore, genetic paralysis was less detrimental than adherence in PBS. However, to fully assess the effect of PF16 deletion on IFT train speed in free flagella, we need to measure IFT train speed in naturally beating flagella.

### Visualization of IFT trains in free-beating flagella using high-frame-rate, dual-color imaging shows higher train speeds

**Figure S1.**
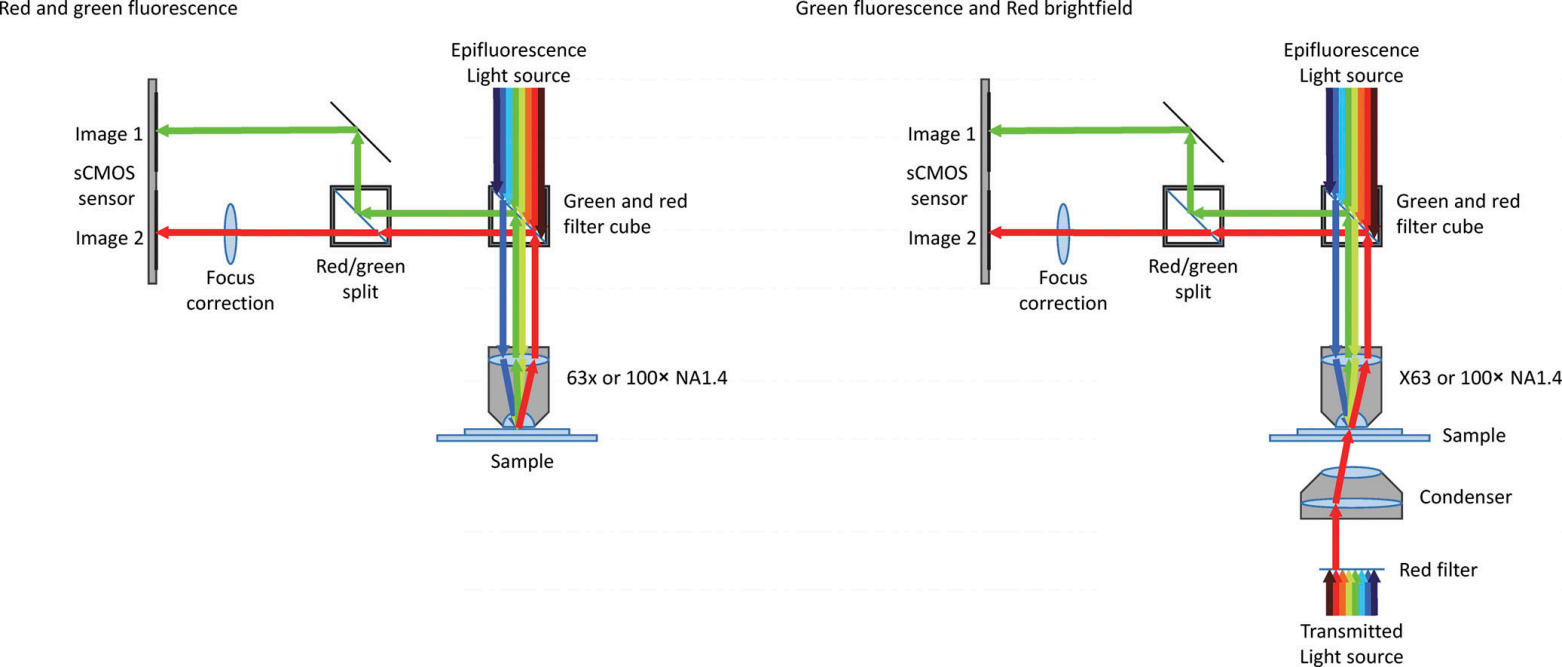
**Schematic diagram of the flexible optical setup utilized in dual-color imaging adapted from Wang et al. (2020).** The transmitted light source can be subjected to a red filter. The epifluorescence is passed through the dual-band 450–490-nm (blue) and 550–590-nm (yellow/green) excitation filter of a custom filter cube (59022bs; Chroma Technology) before reaching the sample. The dual-band (green and red) dichroic mirror of the custom filter cube reflects green (500–545 nm) and red (600–660 nm) light emitted from the sample into the optical splitter. Here, a 565-nm dichroic filter (T565lpxr; Chroma Technology) splits the light through a red transmitted (Chroma Technology, ET632/60 m) or green reflected (ET520/40 m; Chroma Technology) light filter according to wavelength. These two light paths are reflected by mirrors and projected in parallel onto the camera sensor.

**Figure 2.**
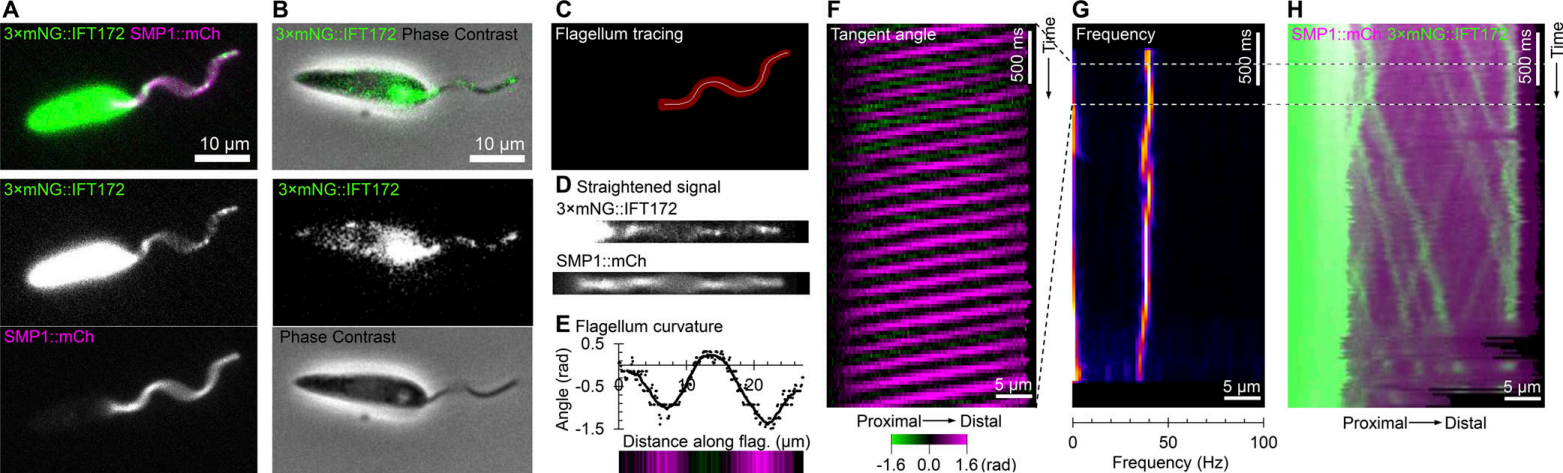
**Workflow for quantitative IFT analysis in beating flagella. (A)** Still frame from a 200 Hz dual-color, high-frame-rate video micrograph of a cell expressing 3×mNG::IFT172 (IFT marker) and SMP1::mCh (flagellar membrane marker). **(B)** Still frame from a 200 Hz dual-color, high-frame-rate video micrograph of a cell expressing 3×mNG::IFT172 (IFT marker) in fluorescence and phase contrast. **(C)** SMP1::mCh image used for flagellum tracing (red) and the flagellum midline (white line) determined by thresholding. **(D)** Digitally straightened view of the flagellum showing the same frame as in A straightened using the midline in B. **(E)** Tangent angle at different distances along the flagellum, represented as a graph and a color-coded bar, showing the same frame as in A. **(F)** Kymograph of flagellum tangent angle over time for a 100-frame section of the dual-color, high-frame-rate video micrograph. **(G)** Power spectrum over time, calculated from the flagellum tangent angle kymograph, for the full-length video micrograph. **(H)** Kymograph of 3×mNG::IFT172 and SMP1::mCh fluorescent signal over time for the full-length video micrograph. IFT trains are readily visible. Animated version of A and D–H in Video 2.

Analyzing IFT in live swimming cells requires simultaneous visualization of tagged IFT fluorescence and flagellum position. We used dual-color widefield epifluorescence microscopy with filters, dichroic mirrors, and a beam splitter to visualize either red and green fluorescence or green fluorescence and red phase contrast simultaneously on a single camera (Wang et al., 2020), capturing short (4.75 s) high-frame-rate (100 or 200 Hz) videos (Fig. S1). Flagellum position was readily visible using cell lines expressing 3×mNG::IFT172 alone using red-light phase contrast or 3×mNG::IFT172 and SMP1::mCh using red fluorescence (Fig. 2, A and B). The flagellum was automatically traced from the red signal (Fig. 2 C) and then used to digitally straighten 3×mNG::IFT172 signal from each video frame (Fig. 2 D), and the flagellum coordinates (Fig. 2 E) were used to generate tangent angle kymographs representing flagellum curvature changes over time (Fig. 2 F). We further summarized changes in flagellum curvature as a beat frequency spectrum over time (Fig. 2 G) (Walker et al., 2019a) for comparison with the kymograph of IFT (Fig. 2 H and Video 2). Anterograde and retrograde IFT train speeds in flagella free to beat were higher than in adhered flagella, reaching speeds similar to *Chlamydomonas* (Chien et al., 2017) and *T. brucei* (Santi-Rocca et al., 2015) immobilized flagella IFT train speed.

**Video 2.** **The generation of IFT and tangent angle kymographs from an example 3×mNG::IFT172 SMP1::mCh** **cell.** This video extends Fig. 2. Dual-color widefield epifluorescence microscopy was used to visualize red and green fluorescence at 200 Hz. Video micrograph and digitally straightened cell are shown with a white line tracing down the IFT and tangent angle kymograph with time in milliseconds displayed to show how IFT and tangent angle kymographs are generated from straightened flagellum and image sequence.

**Figure S2.**
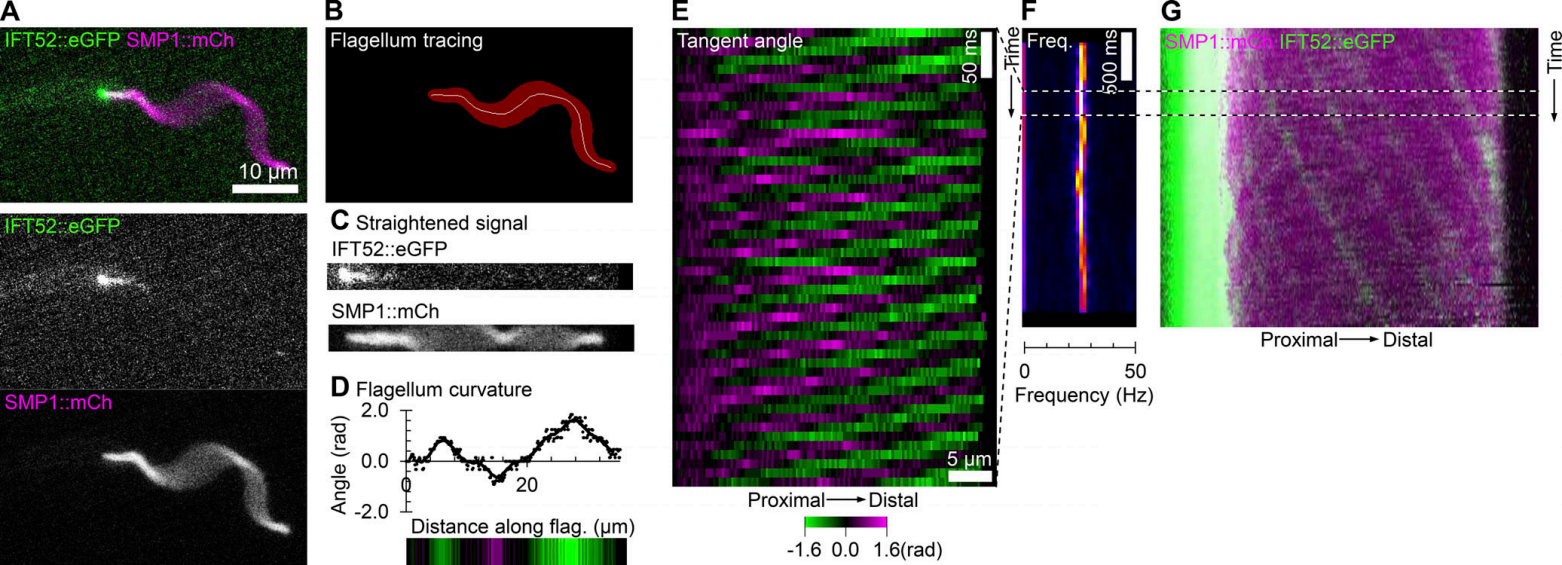
**Digital straightening allows reconstruction of IFT in beating flagella even when IFT particles are not directly visible. (A)** Still frame from a 100-Hz dual-color, high-frame-rate video micrograph of a cell expressing IFT52::eGFP (IFT marker) and SMP1::mCh (flagellar membrane marker). Individual IFT particles are not visible in the flagellum and flagellar motion is significantly blurred at this exposure time (10 ms). **(B)** SMP1::mCh image used for flagellum tracing (red) and the flagellum midline (white line) determined by thresholding. **(C)** Digitally straightened view of the flagellum, showing the same frame as in A straightened using the midline in B. **(D)** Tangent angle at different distances along the flagellum, represented as a graph and a color-coded bar, showing the same frame as in A. **(E)** Kymograph of flagellum tangent angle over time for a 50-frame section of the dual-color, high-frame-rate video micrograph. **(F)** Power spectrum over time, calculated from the flagellum tangent angle kymograph, for the full-length video micrograph. **(G)** Kymograph of IFT52::eGFP and SMP1::mCh fluorescent signal over time for the full-length video micrograph. Animated version of A and C–G in Video 3.

Having optimized this method, we were able to generate IFT kymographs even for IFT52::eGFP signal in 100 Hz videos. Here, IFT trains are rarely visible in individual frames. However, multiple straightened frames can be summed to increase signal making some bright anterograde IFT trains visible (Fig. S2 and Video 3).

**Video 3.** **Generation of IFT and tangent angle kymographs in beating flagellum even when IFT particles are not directly visible.** This extends Fig. S2. Dual-color video micrograph taken at 100 Hz of a cell expressing IFT52::eGFP (IFT marker) and SMP1::mCh (flagellar membrane marker). Digitally straightened flagellum and its six rolling frame average is shown for each frame with a white line tracing down the IFT and tangent angle kymograph with time in milliseconds displayed to show how IFT and tangent angle kymographs are generated from straightened flagellum and image sequence.

### IFT train speed is independent of flagella beat type

**Figure 3.**
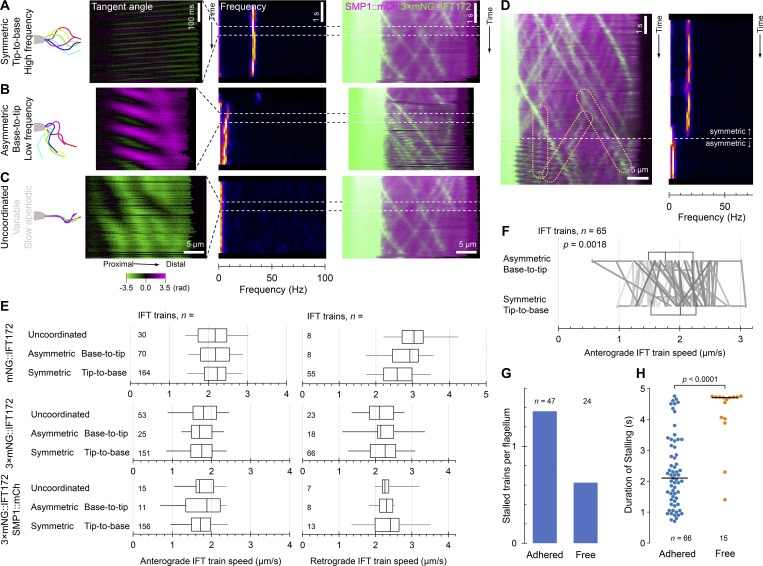
**Anterograde and retrograde IFT speed is largely independent of flagellar beat type. (A–C)** Examples of IFT trains traveling in flagella undergoing different beat types. In each panel, from left to right: cartoon of one cycle of the beat, tangent angle kymograph, beat power spectrum over time, kymograph of 3×mNG::IFT172 and SMP1::mCh fluorescent signal. **(A)** A flagellum undergoing a symmetric tip-to-base beat. **(B)** A flagellum undergoing an asymmetric base-to-tip beat. **(C)** A flagellum undergoing uncoordinated slow aperiodic motion. **(D)** Kymograph of 3×mNG::IFT172 and SMP1::mCh fluorescent signal and power spectrum over time for a flagellum switching from symmetric tip-to-base to asymmetric base-to-tip beating. Example anterograde, retrograde, and stalled IFT trains that were part-way along the flagellum during the switch are highlighted. **(E)** Box plots of the speed of anterograde and retrograde IFT trains in the flagella of mNG::IFT172, 3×mNG::IFT172, and 3×mNG::IFT172 SMP1::mCh based on mNG signal in moving flagella undergoing different beat types. The box represents the lower quartile, median, and upper quartile. The whiskers mark the fifth and 95th percentile. There were no significant differences (P > 0.05, ANOVA) in anterograde or retrograde IFT train speed in flagella undergoing different beats. **(F)** Paired analysis of individual anterograde trains where beat type switched, pooling data from 3×mNG::IFT172 and 3×mNG::IFT172 SMP1::mCh. Box and whiskers represent statistics as in E, lines represent paired data points, P value from a two-tailed paired *t* test. **(G)** Bar chart of the mean number of stalled trains per flagellum, analyzed from the first 4.75 s of videos of adhered or free flagella. *n* is the number of flagella analyzed. **(H)** Bee swam chart of the duration of stalled trains in adhered and free 3×mNG::IFT172 flagella. Each dot represents a stalled train, traced from the *n* flagella stated in G. The P value of a Mann–Whitney *U* test between the two samples is presented.

In addition to the high-frequency symmetric tip-to-base beat, *Leishmania* also undergo low-frequency base-to-tip asymmetric beats and uncoordinated aperiodic movement (Fig. 3, A–C). Tracking individual anterograde and retrograde IFT trains, we never observed a clear change in speed associated with a change in beat type (Fig. 3 D).

We quantified this two ways. First, a blinded analysis, where trains were identified, traced, and speed calculated, and then independently mapped to flagellum beat type. There was no significant difference in the speed of anterograde or retrograde IFT trains between beat types (Fig. 3 E). Second, a paired measurement of train speed before and after a switch in beat type, only considering the more readily visible anterograde trains. To calibrate IFT train speed decrease over time, we made paired measurements of individual train speeds early and late in videos of flagella undergoing a constant symmetric beat, measuring a mean decrease rate of 0.071 μm/s^2^ (*n* = 58). Paired measurements before and after a change between symmetric and asymmetric beat, corrected for slowdown, showed slightly but significantly slower train speed in flagella undergoing an asymmetric beat (Fig. 3 F).

IFT train speed may be weakly dependent on naturally occurring beat type but is not strongly coregulated. Intraflagellar secondary messengers controlling beat type seem unlikely to also regulate IFT speed, and flagella curvature and direction of wave propagation do not cause large spatial hinderance to IFT trains. Perhaps IFT trains are restricted to particular doublet tracks, like in *T. brucei* (Bertiaux et al., 2018), that are not affected by planar flagellar bending. *Leishmania* life stages have characteristic flagellum length and swimming behaviors (e.g., metacyclic: fast-swimming, long flagellum [Sacks and Perkins, 1984; Findlay et al., 2021]; and amastigote: immotile, short flagellum [Gluenz et al., 2010; Gull, 2009]). A 10% change in IFT train speed due to beat-type switches to allow chemotaxis seems unlikely to directly cause these morphological changes by modulating IFT.

### IFT train stalling occurs in beating flagella

IFT trains can stall, sometimes with functional consequences. Surface-adhered *Chlamydomonas* can undergo gliding motility, which we have never observed in *L. mexicana,* where IFT-carrying transmembrane proteins interact through the flagellar membrane with the substrate to stall train movement or drive gliding (Bloodgood and Salomonsky, 1989; Shih et al., 2013; Collingridge et al., 2013). We observed that IFT train stalling occurs in some freely beating flagella. Comparing stalled trains in the first 4.75 s of IFT kymographs from adhered flagella to the 4.75-s-long free flagella videos showed twice as many stalled trains per flagellum when adhered (Fig. 3 G). However, stalls tended to be longer (often the full 4.75 s) in free flagella (Fig. 3 H). It is notable that IFT trains can stall, albeit less frequently, without interaction with a solid substrate.

### Anterograde IFT train speed increases in flagella with paralyzed or reduced frequency beating

**Figure S3.**
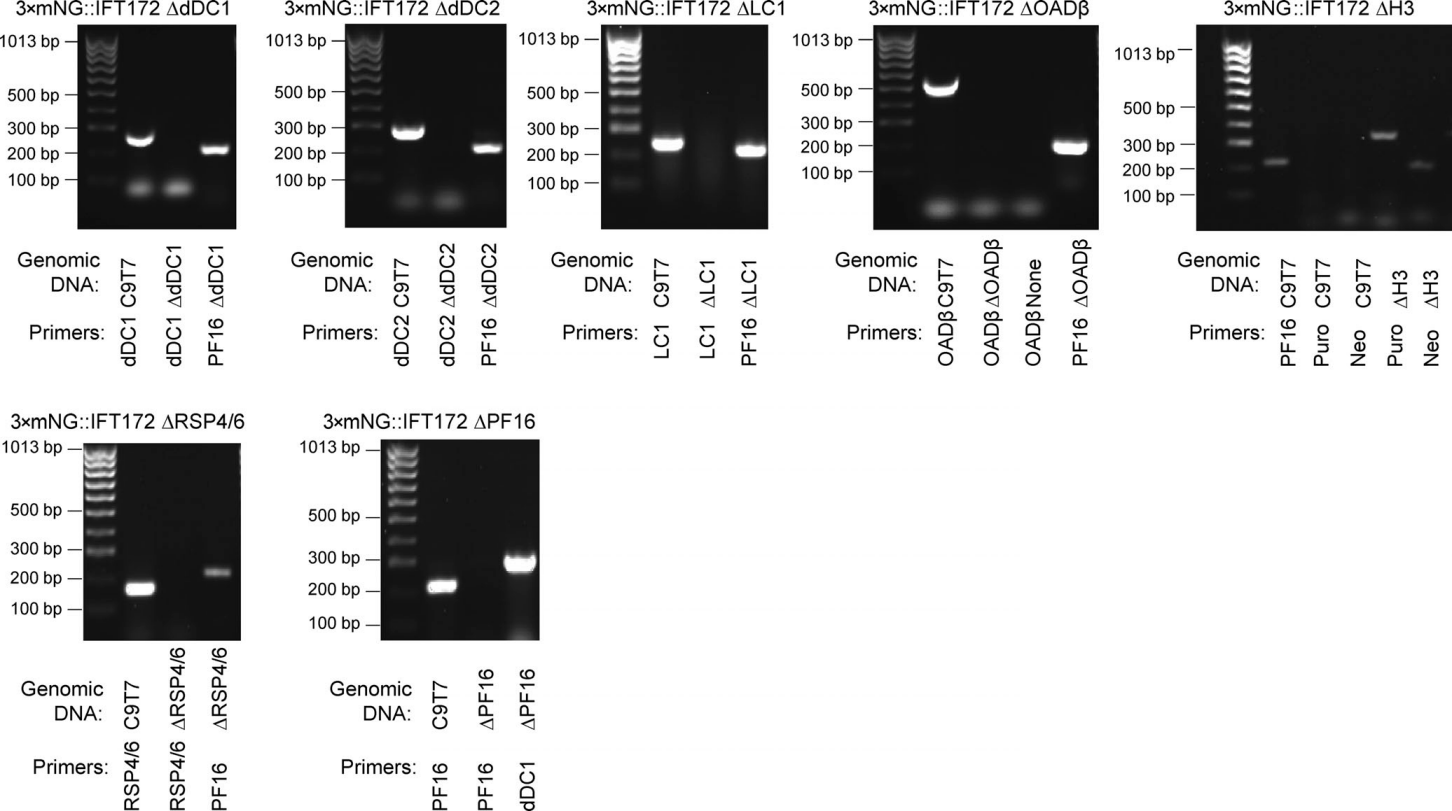
**PCR confirmation of target ORF deletion.** Images of PCR products visualized by agarose gel electrophoresis. For each PCR, the genomic DNA and the ORF to which the primers anneal are indicated. In general, one test sample, one test sample with primers to an unmodified locus. For 3×mNG:IFT172 ΔH3, positive validation for the presence of double drug markers was used as H3 gene repeats have identical coding and upstream regions, and drug marker insertions were required negative control for deletion mutants. LC4-like deletion cell line is from Edwards et al. (2018). Source data are available for this figure: SourceData FS3.

Surprisingly, anterograde IFT train speed in adhered 3×mNG::IFT172 was slower than in adhered and genetically paralyzed 3×mNG::IFT172 ΔPF16 (Fig. 1 B). To test if permanent perturbations to flagellar movement would generally affect IFT, we generated a panel of cell lines expressing 3×mNG::IFT172 with deletions of genes with known (PF16, dDC2) or predicted (dDC1, RSP4/6, OADβ, LC1) importance for normal flagellar beating in *Leishmania* (Fig. S3 and Table S1).

**Figure 4.**
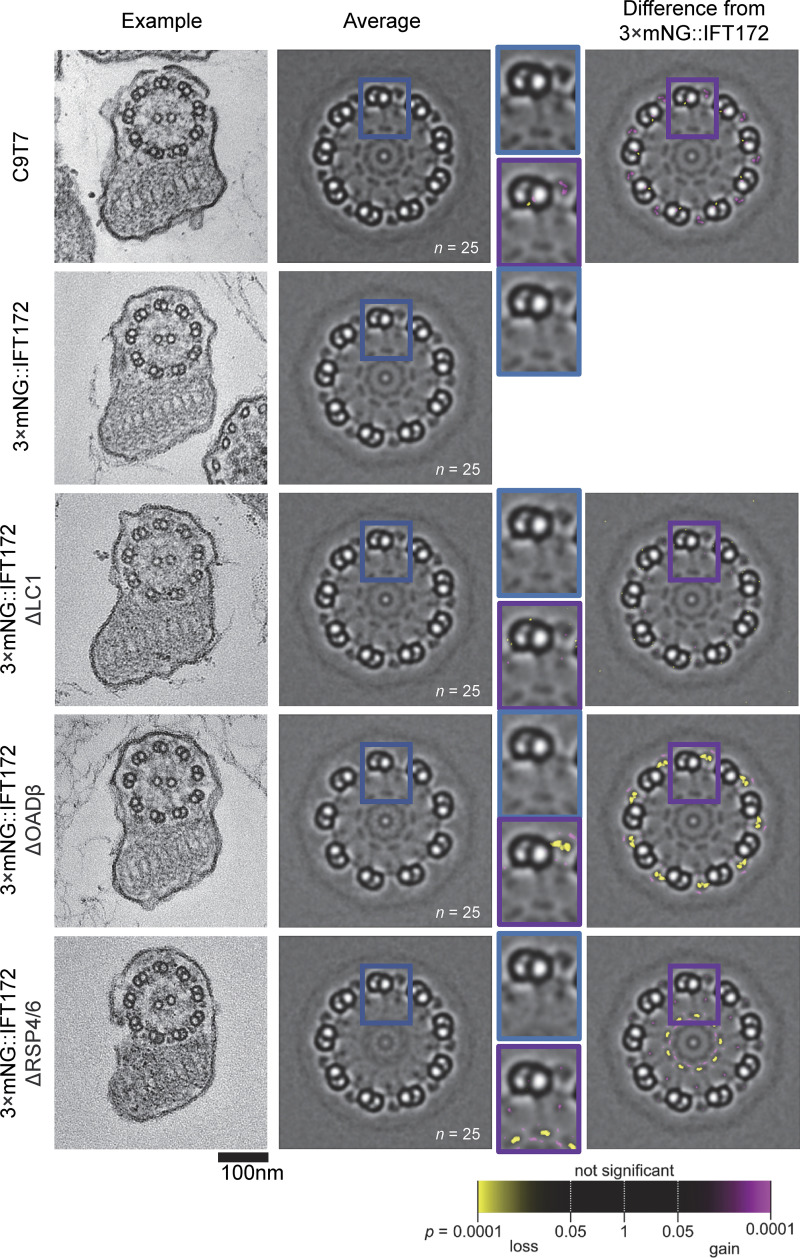
**Axoneme structure changes in deletion mutants with decreased swimming speed.** Transmission electron micrographs of cross-sections of the axoneme and paraflagellar rod showing the change in ultrastructure in deletion mutants. From left to right: column one, representative example transverse section through the flagellum showing the axoneme and paraflagellar rod. Column two, an average of 25 ninefold rotationally averaged axonemes. The region in the blue box is shown enlarged to the right to show one microtubule doublet and associated complexes. Column three, comparison of electron density to 3×mNG::IFT172, using a per-pixel Mann–Whitney *U* test. The region in the purple box is shown enlarged to the left to show one microtubule doublet.

**Figure 5.**
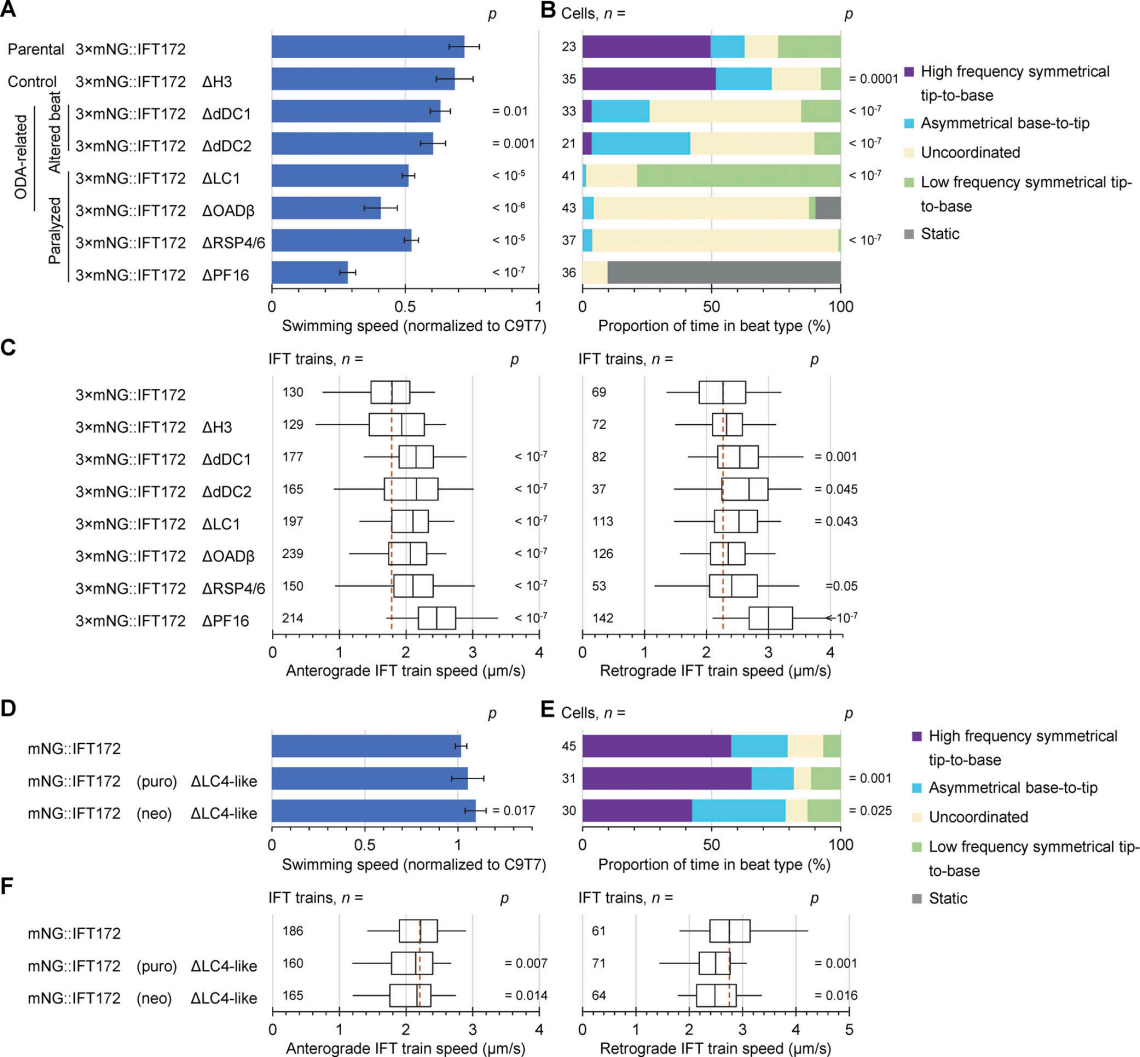
**Anterograde IFT speed increases in deletion mutants with decreased flagellar movement, and retrograde IFT train speed is decreased in deletion mutants with increased flagellar movement. (A–C)** Characterization of slower-swimming mutants, generated from the 3×mNG::IFT172 parental cell line. **(D–F)** Characterization of faster-swimming mutants, generated by IFT17 tagging in the ΔLC4-like cell line. **(A and D)** Bar chart of the mean swimming speed of deletion mutants. Error bars plotted show the standard deviation of six replicates. Each replicate is the mean swimming speed of one cell line sample, normalized to a sample of the C9T7 cell line (the parental cell line) captured on the same day. For each replicate, over 1,000 cells were analyzed. P values for significant changes (two-tailed *T* test in comparison to parental, P < 0.05) are shown. **(B and E)** Stacked bar graph representing the proportion of time spent in different beat types for each cell line. P values for significant changes (chi-squared test, P < 0.05) are shown, except ΔPF16 and ΔODAβ, where a chi-squared test is not valid as static flagella were never observed in the parental line. **(C and F)** Boxplots of the speed of anterograde and retrograde IFT trains in the parental and the motility-deficient deletion mutant cell lines. The box represents the lower quartile, median, and upper quartile. The whiskers mark the 5th and 95th percentile. The brick-red dashed line shows the median IFT train speed for anterograde and retrograde trains in the control tagged IFT172 cell line (two-tailed *T* test in comparison to control, P < 0.05).

For the previously uncharacterized RSP4/6, OADβ, and LC1 deletions, we confirmed the predicted axoneme defects by EM (Fig. 4). For each deletion cell line, we confirmed the expected effect on cell swimming speed (Fig. 5 A) and then analyzed the beat type (Fig. 5 B) and IFT train speed (Fig. 5 C). Categorization of cells by time undergoing normal (symmetric, asymmetric, or uncoordinated beats) or mutant-specific (low-frequency symmetric tip-to-base beat or paralyzed) (Fig. 5 B) broadly replicated previous characterization (Beneke et al., 2019; Gadelha et al., 2007; Wheeler, 2017). However, for flagellum tracing, the cell must stay precisely in focus in the field of view and the flagellum must not fold back and cross itself, resulting in some biases. Finally, we measured anterograde and retrograde IFT train speed in flagella free to beat (if able) (Fig. 5 C).

First, we analyzed ΔdDC1 and ΔdDC2. As previously shown, ΔdDC2 underwent less symmetric and more asymmetric beating due to loss of distal ODAs (Edwards et al., 2018) and swam slower (Fig. 5, A and B). dDC1 is expected to form a heterodimer with dDC2, and, as expected, ΔdDC1 phenocopied ΔdDC2. Intriguingly, anterograde and retrograde IFT train speed was increased in both (Fig. 5 C). To confirm that drug-selectable markers for gene deletion were not spuriously affecting IFT train speed, we deleted one histone 3 (H3) gene from the H3 tandem array using the same markers. Swimming speed and IFT train speeds were indeed unchanged (Fig. 5, A and C). As IFT train speed upon naturally occurring switches from symmetric to asymmetric beats showed a potential small decrease (Fig. 3, E and F), the increased train speed in asymmetric beat-preferring ΔdDC1 and ΔdDC2 is not likely to be directly due to flagellar geometry associated with the tightly curved asymmetric waveform.

To dissect this effect, we used ΔLC1 and ΔOADβ. In *Chlamydomonas*, OADβ is required for ODA assembly (Kamiya, 1988; Liu et al., 2008; Mitchell and Rosenbaum, 1985) and OADβ and LC1 are both required for normal beating (Sakato-Antoku and King, 2023). In *T. brucei*, both OADβ and LC1 are required for both ODA assembly and normal flagella beating (Baron et al., 2007; Ralston et al., 2011). Our EM showed LC1 was not necessary for *L. mexicana* ODA assembly while OADβ was, similar to *Chlamydomonas* (Fig. 4). ΔLC1 gave a disrupted beat but did not replicate the reversed beat direction described in *T. brucei* (Baron et al., 2007) (Fig. 5 B). Given these results, ΔLC1 and ΔOADβ provide insight into whether the change in beat type or a loss of ODAs causes increased IFT train speed—if purely an effect of disrupted beating ΔOADβ and ΔLC1 should phenocopy ΔdDC1 and ΔdDC2, but if an effect of missing ODAs ΔLC1 should have normal train speed. ΔOADβ and ΔLC1 both swam slower, had increased uncoordinated flagella movement, and had increased anterograde IFT train speed (Fig. 5, A–C). Therefore, increased IFT train speed is unlikely to be due to missing ODAs.

To test the effect of flagellar paralyzation by disruption of non-ODA structures, we used ΔPF16 and ΔRSP4/6. In *Chlamydomonas,* RSP4 and RSP6 are necessary for RS head assembly (Wei et al., 2010), and ΔRSP4/6 caused loss of RS heads in *L. mexicana* (Fig. 4). PF16 is necessary for central pair assembly, previously shown in several species (Espada et al., 2021; Martel et al., 2017; Yagoubat et al., 2020; Dutcher et al., 1984) including *L. mexicana.* These are related mutants as a charge-based interaction between the RS head and central pair complex is proposed to detect axoneme mechanical strain (Grossman-Haham et al., 2021). Both had the expected severe swimming defects (Beneke et al., 2019), RSP4/6 necessary for a coordinated beat and PF16 necessary for any flagellum movement, without affecting outer doublet decorations near where IFT trains travel (Fig. 5 B). Both had increased anterograde IFT train speed, strongest for ΔPF16 (Fig. 5 C). Therefore, a permanent reduction in swimming speed and flagella motility increases IFT train speed, significantly increasing retrograde speed in some and anterograde speed in all cell lines (Fig. 5, A–C).

### Retrograde IFT train speed decreases in flagella with increased beat frequency

As IFT train speed broadly inversely correlated with reduced cell swimming speed and the propensity of flagella to beat (Fig. 5 C), we tested the inverse, using the distal ODA-associated protein LC4-like. ΔLC4-like has a higher frequency symmetric beat (Edwards et al., 2018). Unfortunately, we were unable to delete LC4-like in the cell line expressing 3×mNG::IFT172, thus tagged IFT172 with mNG in an existing ΔLC4-like line, generating two cell lines using different drug selectable markers (puro and neo). Consistent with an increased beat frequency, both ΔLC4-like lines swam slightly faster with broadly normal waveform types (Fig. 5, D and E). Both mNG::IFT172 (puro) and mNG::IFT172 (neo) ΔLC4-like had significantly higher flagella beat frequency at 33.3 ± 7.3 Hz (*n* = 23, mean ± standard deviation, P = 0.02, two-tailed *t* test) and 31.6 ± 7.7 Hz (*n* = 27, P = 0.02, two-tailed *t* test), respectively, compared with 27.0 ± 8.4 Hz for mNG::IFT172 (*n* = 39). In both, anterograde and retrograde IFT trains were significantly slower than the mNG::IFT172 control, with retrograde being most reduced (Fig. 5 F). This indicates an inverse relationship between IFT train speed and flagellum beat frequency.

Overall, this suggests competition of IFT and axoneme motor proteins for ATP. If altered train speed was a spatial access effect, ΔOADβ (lacking all ODAs) and ΔdDC1 and ΔdDC2 (lacking distal ODAs) should have similar train speed phenotypes, while ΔLC4-like and ΔLC1 (lacking one small ODA component) should have similar phenotypes. Instead, IFT train speed inversely correlated with flagellar movement, being fastest in ΔOADβ and ΔLC1 (which cannot beat), fast in ΔdDC1 and ΔdDC2 (a low-frequency altered beat), and slowest in ΔLC4-like (which beat faster).

### Conclusions

Using dual-color epifluorescence microscopy, we showed that IFT in motile flagella in their physiological beating state is broadly similar to immobilized flagella, but with faster (Fig. 1 B versus Fig. 3 E) and fewer stalled (Fig. 3, G and H) IFT trains. We showed that train speed is not strongly affected by natural switches in flagellar beat, tentatively observing a decrease with asymmetric beating (Fig. 3, E and F), but deletion mutants with perturbed beating had a clear inverse correlation of train speed with flagellum beating (Fig. 5, C and F).

This highlights issues with analyzing IFT in mechanically or genetically immobilized flagella. Adherence of flagella to a slide had a strong time-dependent detrimental effect, particularly for retrograde IFT trains (Fig. 1, C–E), decreasing the sensitivity of this methodology to small changes in IFT train and indicating experimental design must minimize immobilization time. Genetic paralysis of flagella also had a confounding effect—an increase in IFT train speed (Fig. 1), which nonetheless decreased over observation time (e.g., Fig. 1 B versus Fig. 5 C for ΔPF16).

From the broadly inverse correlation of IFT train speed and rapidity of flagellum movement in deletion mutants (Fig. 5, C and F), we suggest there may be competition for ATP as an energy source. While we did not measure axonemal ATP usage, flagella that are unable to beat normally (ΔPF16, ΔRSP4/6, ΔLC1) are unlikely to have normal usage, and mutants missing most of their ODAs (ΔOADβ, ΔdDC1, and ΔdDC2) must have dramatically reduced capacity for usage.

This might not be a universal phenomenon, and we have only analyzed one species. For example, the *Chlamydomonas* beat is higher frequency, thus competition for ATP may be more pronounced. Interestingly, in mechanically immobilized flagella of *Chlamydomonas* with temperature-sensitive paralysis, IFT train speed increases around the temperature at which paralysis occurs (Iomini et al., 2001), which is consistent with alleviated competition for ATP. Conversely, in *Chlamydomonas*, flagellar-localized glycolytic enzymes may represent a further ATP source (Mitchell et al., 2005). Trypanosomatid flagella similarly contain orthologs of glycolytic enzymes, however they are likely inactive (Brown et al., 2014). Trypanosomatids do, however, use arginine phosphate as a high-energy phosphate shuttle (Ooi et al., 2015). However, fundamentally, all energy sources—ATP, glucose, or phosphate shuttles—face the same restriction of diffusing into the flagellum from its base.

Our work demonstrates the power of analyzing IFT in flagella undergoing their natural beating behavior, but open questions remain: Does immobilization by high viscosity have a consistent effect? Why were anterograde and retrograde trains differentially affected in fast- and slow-beating flagellar mutants? What is the impact of mechanical or genetic immobilization on IFT train frequency and cargo? Our technology development now allows analysis of the interplay between IFT and beating in dynamic situations such as during flagella assembly and disassembly, in life cycle transitions, and in different organisms.

## Materials and methods

### *L. mexicana* cell culture

*L. mexicana* (MNYC/BZ/62/M379) procyclic promastigotes stably expressing Cas9 and T7 RNA polymerase (Cas9 T7) (Beneke et al., 2017) were cultured at 28°C in an M199 medium (Life Technologies), supplemented with 10% fetal calf serum, 0.005% haemin, and 5 mM HEPES (pH 7.4) and kept at cell densities permitting exponential growth (10^5^–10^7^ cells/ml).

### *L. mexicana* genetic modification

*L. mexicana* Cas9 T7 promastigotes were genetically modified using CRISPR-based genome editing (Beneke et al., 2017; Beneke and Gluenz, 2019). These and derived cell lines were maintained under 32 μg/ml hygromycin B and 50 μg/ml nourseothricin sulfate (Cambridge Bioscience Ltd).

The cell line IFT52::eGFP SMP1::mCh was the same as used in Wheeler et al. (2015). Endogenous tagging of IFT172 (LmxM.21.0980) used constructs generated by long primer PCR using the pLPOT plasmid series (Edwards et al., 2018) as template DNA, where the long primers introduce flanking homology arms to a fluorophore and drug-selectable marker encoded in the template plasmid (Dean et al., 2015). For endogenous tagging with mNG, we used pLPOT mNG Puro or pLPOT mNG Neo, and for tagging with 3×mNG, we generated pLPOT 3×mNG Blast by cloning 3×mNG from pPOT 3×mNG (Paterou et al., 2023, *Preprint*). single guide DNA (sgDNA) encoding the CRISPR sgRNA and a T7 polymerase promoter was generated by PCR (30 cycles of 30 s at 94°C, 30 s at 60°C, and 30 s at 72°C) with one primer encoding the gene-specific protospacer adjacent motif site and T7 promoter, and the other encoding the remainder of the sgRNA. Primer sequences were designed using LeishGEdit, and PCR was carried out as previously described (Beneke et al., 2017; Dean et al., 2015): primers, template plasmid, and PCR mix for 3 min at 95°C, then 35 cycles of 15 s at 94°C, 30 s at 65°C, and 60 s at 72°C, and 1 min final elongation at 72°C. Deletion of both alleles of H3 (LmxM.10.0990), dDC1 (LmxM.15.0540), LC1 (LmxM.24.1030.1), OADβ (LmxM.13.1650), RSP4/6 (LmxM.13.0430), or PF16 (LmxM. 20.1400) also used constructs generated by long primer PCR, using pT Puro or pT Neo (Beneke et al., 2017) as a template, and sgDNAs generated by PCRs (33 s at 94°C, 35 cycles of 15 s at 94°C, 30 s at 65°C, and 60 s at 72°C, then 2 min at 72°C). All cell lines generated are listed in Table S1 and all primer sequences are listed in Table S2.

Successfully modified cell lines were selected and then maintained using the necessary combination of 5 μg/ml blasticidin, 40 μg/ml neomycin, and 20 μg/ml puromycin (Cambridge Bioscience Ltd) for the blasticidin-S deaminase (Blast), neomycin-kanamycin phosphotransferase (Neo), and puromycin N-acetyltransferase (Puro) drug-selectable markers, respectively, in addition to hygromycin and nourseothricin. Following drug selection, genomic DNA was extracted from mutant deletion cell line using a DNeasy (Qiagen) kit as per the manufacturer’s instructions. Three PCRs (3 min at 95°C, then 35 cycles of 15 s at 95°C, 15 s at 58°C, and 30 s at 72°C, then 1 min final elongation at 72°C) were used to validate deletion: (1) PCR with C9T7 (parental) gDNA and primers in the deleted ORF, to confirm product can be generated; (2) PCR with deletion cell line gDNA and primers in the deleted ORF, to confirm that the ORF is absent; (3) PCR with deletion cell line gDNA and primers in control ORF PF16 for all except in the PF16 deletion mutant cell line where dDC2 was used, to confirm that the gDNA can be used as a template. Primers for deletion validation were Primer3 designed, exactly as listed in Beneke et al., 2019. Presence or absence of the PCR product was confirmed by agarose gel electrophoresis, shown in Fig. S3.

If necessary, cell lines underwent subcloning in 96-well plates, with 200 µl per well at 0.5 cells/ml. Cells were taken from positive wells and transferred to flasks before being validated.

### Fluorescence microscopy

For all IFT visualization, *L. mexicana* were imaged live and 1 ml samples were taken from cultures in the late exponential growth phase, 5 × 10^6^ to 1 × 10^7^. Microscopy was carried out at ambient temperature (≈22°C) using an Axio Observer A1 (Zeiss) microscope with a 100× or 63× NA 1.40 Ph3 objective.

For adhered samples, cells were washed thrice in PBS to enhance adhesion to the slide. The cells were resuspended in 50 μl of PBS and 1 μl was placed on a slide and a coverslip applied. This led to cell and flagella adhesion to the slide. The mechanically immobilized cells were then imaged at 2.5 Hz for IFT52::eGFP and 5 Hz for other cell lines for 512 frames using a Hamamatsu Flash 4 camera using Zeiss Zen-2.6 (Blue Edition).

For swimming samples, 1 ml of cells were taken from culture and centrifuged, then resuspended in 100 μl of M199 medium to increase cell density. High-frame-rate, dual-color imaging was completed using a red filter (ThorLabs Astronomy red) on phase contrast, custom filter cubes (Chroma), and an Optosplit II (Cairn Research) optical splitter projecting to an Andor Neo 5.5 Camera using Andor Solis version 4.22.3. The custom interference cube had a dual pass (blue and yellow) excitation filter (59022x; Chroma Technology) and dual pass dichroic mirror (59022bs; Chroma Technology) for transmitting green and red wavelengths from the sample to the optical splitter. In the Optosplit II, a cube installed with a 565-nm dichroic filter (T5651pxr; Chroma Technology) directed light through their respective green reflected (ET520/40m; Chroma Technology) and red transmitted light filters (ET63260m; Chroma Technology). For simultaneous dual-wavelength fluorescence imaging, two light paths were projected onto the single camera sensor in parallel. This optical setup and its light pathway have previously been described (Wang et al., 2020), except here we used a red filter instead of green as graphically presented in Fig. S1.

Image analysis for both adhered and swimming samples was carried out using ImageJ Version 1.53k (Schneider et al., 2012). The flagella of adhered cells were traced manually and the coordinates were used to straighten the flagella mNG IFT signal; from the straightened IFT fluorescence, the fluorescence intensity at each distance along the flagellum per frame is plotted to generate IFT kymographs.

For swimming cells, reference images of cells were captured with white-light phase contrast to get a phase contrast in the red and green channels. These were used to measure the relative position of the two channels to the nearest pixel. Next, using these offsets, a composite containing the aligned channels from dual-color videos was generated. To automatically trace the flagellum, the red-light phase contrast or red flagellar membrane channel underwent intensity thresholding, and the midline of the flagellum was identified by skeletonization. This methodology is as previously described (Walker and Wheeler, 2019). Using a custom ImageJ script, swimming cells were automatically traced in each frame, and the coordinates were used to digitally straighten the IFT fluorescence using the ImageJ selection straighten tool. From the traced flagella coordinates, the tangent angle relative to the flagellum base was calculated at each distance along the flagellum per time frame and then plotted per frame to generate a tangent angle kymograph. This is as previously described (Walker and Wheeler, 2019; Wang et al., 2020). We calculated a beat power spectrum by Fast Fourier transform using a rolling window of 128 frames to display change in beat frequency over time. This is as previously described (Walker et al., 2019b). IFT kymographs were generated for adhered flagella from a straightened IFT signal. Together, these showed flagella bending and IFT fluorescence signal along the flagella over time, respectively.

For each tangent angle kymograph, polygons representing the region of the flagellum undergoing the same flagellum movement were selected using the polygon tool, recorded, and categorized as one of five beat types: symmetric tip-to-base, asymmetric base-to-tip, uncoordinated, low-frequency symmetric tip-to-base, or static. Separately, using the line tool, anterograde and retrograde IFT trains were marked in the IFT kymograph and recorded. Then, using an automated script, the speeds of each IFT train were calculated from the distance traveled over time taken to do so and then mapped to the respective beat type for that time and position along the flagellum.

### Transmission EM

2.5% glutaraldehyde (glutaraldehyde 25% stock solution, EM grade; Electron Microscopy Sciences) was used to fix *L. mexicana* in medium at room temperature for 10 min. 10 ml of fixed *L. mexicana* in media was centrifuged at 16,000 *g* for 5 min and supernatant was discarded. 0.1 M cacodylate buffer (pH 7.2) with 2.5% glutaraldehyde and 4% PFA (16% stock solution, EM grade; Electron Microscopy Sciences) was added to fix the pellet for a minimum of 2 h. Cells were stained with OsO_4_ (1%, from 4% stock aqueous solution; Taab Laboratories Equipment). Samples underwent ethanol solution–derived serial dehydration prior to embedding and polymerization for 24 h at 60°C in low-viscosity resin Agar 100 (Agar Scientific). A Leica EM UC7 ultramicrotome was used to cut 80–90-nm ultrathin sections, placed on nickel grids, and stained with uranyl acetate (1%, wt/vol) (uranyl acetate dihydrate; Electron Microscopy Sciences) for 5 min at room temperature and stained with lead citrate (80 mM Reynolds solution; Reynolds, 1963) for 5 min. Observations were made on a JEOL 2100 Plus 200 kV transmission electron microscope with a Gatan OneView camera.

For the generation of averaged axoneme views, points in the center of each outer doublet A tubule were marked, fitted to an ellipse, perspective corrected to make this ellipse circular and of a standard size, and then ninefold rotationally averaged. This methodology is as previously described (Gadelha et al., 2006). 25 rotationally averaged axonemes were then aligned using the StackReg plugin for ImageJ and averaged. Difference maps were generated by comparison of the 25 rotationally averaged 3×mNG::IFT172 parent compared to deletion mutants, and per-pixel statistical significance of electron density changes calculated by the Mann–Whitney *U* test (with multiple comparison correction for the number of pixels within the axoneme cross-section).

### Online supplemental material

Supplemental figures contain extended data corresponding to the main figures, a diagram of the optical setup, and validation of deletion mutant cell lines. Fig. S1 is a schematic of the optical configuration used for microscopy of swimming cells. Fig. S2 shows digital straightening and reconstruction of IFT when not directly visible in each frame. Fig. S3 shows gels of validation of deletion mutants by PCR from genomic DNA. Table S1 describes all cell lines used with fluorescent IFT172 (LmxM.21.0980) tag. Table S2 lists primer sequences used in PCR to generate tagging and deletion constructs. Video 1 shows fluorescently tagged IFT trains moving in the flagella immobilized by adherence to glass, paralyzed by PF16 deletion, or both. Video 2 shows the generation of IFT and tangent angle kymographs from an example 3×mNG::IFT172 SMP1::mCh cell. Video 3 shows the generation of IFT and tangent angle kymographs in beating flagellum even when IFT particles are not directly visible.

## Supplementary Material

Table S1 shows tagged and deletion mutant cell lines.

Table S2 shows primer sequences used for protein tagging, gene deletion, and deletion validation.

SourceData FS3 is the source file for Fig. S3.

## Data Availability

All data are available on request.
