# Supplementary material for: Intraflagellar transport speed is sensitive to genetic and mechanical perturbations to flagellar beating
Source: J Cell Biol. 2024 Jun 3;223(9):e202401154. doi: 10.1083/jcb.202401154 (PMC11148470; doi:10.1083/jcb.202401154)
Supplement: Table S1 — shows tagged and deletion mutant cell lines. [file JCB_202401154_TableS1.docx]

| Cell Line Name | Fluorescent tag on IFT172 (LmxM.21.0980) | Tagging Drug Marker | ID of Gene with both alleles deleted | Name of deleted gene | Deletion Drug Marker |
| --- | --- | --- | --- | --- | --- |
| 3×mNG:IFT172 | 3×mNeonGreen | Blasticidin | N/A | N/A | N/A |
| mNG:IFT172 | mNeonGreen | Puromycin | N/A | N/A | N/A |
| 3×mNG:IFT172  ΔH3 | 3×mNeonGreen | Blasticidin | LmxM.10.0990 | Histone 3 | Puromycin & neomycin |
| 3×mNG:IFT172  ΔdDC1 | 3×mNeonGreen | Blasticidin | LmxM.15.0540 | Distal docking complex protein 1 (dDC1) | Puromycin & neomycin |
| 3×mNG:IFT172  ΔdDC2 | 3×mNeonGreen | Blasticidin | LmxM.31.2900 | Outer dynein arm docking complex protein 2 | Puromycin |
| mNG:IFT172 (N)  ΔLC4-like | mNeonGreen | Neomycin | LmxM.01.0620 | Beat Regulation Protein / LC4-like | Blasticidin |
| mNG:IFT172 (P)  ΔLC4-like | mNeonGreen | Puromycin | LmxM.01.0620 | Beat Regulation Protein / LC4-like | Blasticidin |
| 3×mNG:IFT172  ΔLC1 | 3×mNeonGreen | Blasticidin | LmxM.24.1030 | Light Chain 1 | Puromycin & neomycin |
| 3×mNG:IFT172  ΔOADβ | 3×mNeonGreen | Blasticidin | LmxM.13.1650 | Outer Arm Dynein β | Puromycin & neomycin |
| 3×mNG:IFT172  ΔRSP4/6 | 3×mNeonGreen | Blasticidin | LmxM.13.0430 | Flagellar radial spoke protein 4/6 | Puromycin & neomycin |
| 3×mNG:IFT172  ΔPF16 | 3×mNeonGreen | Blasticidin | LmxM.20.1400 | Paralyzed flagella protein 16 | Puromycin & neomycin |

**Supplemental Table 1. Tagged and deletion mutant cell lines.**
