# Supplementary material for: Intraflagellar transport speed is sensitive to genetic and mechanical perturbations to flagellar beating
Source: J Cell Biol. 2024 Jun 3;223(9):e202401154. doi: 10.1083/jcb.202401154 (PMC11148470; doi:10.1083/jcb.202401154)
Supplement: Table S2 — shows primer sequences used for protein tagging, gene deletion, and deletion validation. [file JCB_202401154_TableS2.docx]

| Gene ID | Gene name | Primer Category | Primer Sequence |
| --- | --- | --- | --- |
| LmxM.21.0980 | Intraflagella Transport Protein 172 (IFT172) | Upstream Forward | ACACACACACACACGCACAGAGGTGCCCCTgtataatgcagacctgctgc |
| LmxM.21.0980 | Intraflagella Transport Protein 172 (IFT172) | Upstream Reverse | CATGATGTTCTGGTAAAACTGTACCTGCATactacccgatcctgatccag |
| LmxM.21.0980 | Intraflagella Transport Protein 172 (IFT172) | 5' sgRNA | gaaattaatacgactcactataggGCAACGGTATAGGAGAGAAGgttttagagctagaaatagc |
| LmxM.21.0980 | Intraflagella Transport Protein 172 (IFT172) | Downstream Forward | GGCGCGGCGGCGAGCCCCAACTTCAAGGTGggttctggtagtggttccgg |
| LmxM.21.0980 | Intraflagella Transport Protein 172 (IFT172) | Downstream Reverse | CTTTTTTTATTGGCCCCCTCCCCTTCCCGCccaatttgagagacctgtgc |
| LmxM.21.0980 | Intraflagella Transport Protein 172 (IFT172) | 3' sgRNA | gaaattaatacgactcactataggTCCACGCCCACCCTGCGGGTgttttagagctagaaatagc |
| LmxM.10.0990 | Histone 3 | Upstream Forward | GCCACCACTACCCACCCACCCCACCCACCCgtataatgcagacctgctgc |
| LmxM.10.0990 | Histone 3 | Upstream Reverse | CGCGCGGGCGGTCTCCTTGGTGCGGGACATactacccgatcctgatccag |
| LmxM.10.0990 | Histone 3 | 5' sgRNA | GaaattaatacgactcactataggCGGTGTGGGGGGGAGGGAGTgttttagagctagaaatagc |
| LmxM.10.0990 | Histone 3 | Downstream Forward | CTGGCGCTGCGCCTGCGCGGTGAGCGCCATggttctggtagtggttccgg |
| LmxM.10.0990 | Histone 3 | Downstream Reverse | TCCTGGTTTCACCACTGCTGCACCGCACCAccaatttgagagacctgtgc |
| LmxM.10.0990 | Histone 3 | 3' sgRNA | gaaattaatacgactcactataggTACCCCTCTATCTCTGTCGCgttttagagctagaaatagc |
| LmxM.10.0990 | Histone 3 | Forward | ATCCGCAAGTTCCAGAAGAGCA |
| LmxM.10.0990 | Histone 3 | Reverse | TCCTTCGGCTGGATCGTCAC |
| LmxM.15.0540 | Distal docking complex protein 1 (dDC1) | Upstream Forward | GCACGCACACACACACGGCTGACGCGCACCgtataatgcagacctgctgc |
| LmxM.15.0540 | Distal docking complex protein 1 (dDC1) | Upstream Reverse | GGCGATGTTCTCACTCGCAGTGCCGTTCATactacccgatcctgatccag |
| LmxM.15.0540 | Distal docking complex protein 1 (dDC1) | 5' sgRNA | GaaattaatacgactcactataggGTTTCTTTGTTCCTACGTGTgttttagagctagaaatagc |
| LmxM.15.0540 | Distal docking complex protein 1 (dDC1) | Downstream Forward | CAGCAGCGACGCAACAAGGAAGAAATGGTGggttctggtagtggttccgg |
| LmxM.15.0540 | Distal docking complex protein 1 (dDC1) | Downstream Reverse | AGCTGAGAGCGAGGTTGAGGGGGTGGGGTGccaatttgagagacctgtgc |
| LmxM.15.0540 | Distal docking complex protein 1 (dDC1) | 3' sgRNA | gaaattaatacgactcactataggGGGGGGGGGCAGAGACGCAGgttttagagctagaaatagc |
| LmxM.15.0540 | Distal docking complex protein 1 (dDC1) | Forward | GATGCAAGCGACTATGGCCTTC |
| LmxM.15.0540 | Distal docking complex protein 1 (dDC1) | Reverse | GCAGTTACGCAGCAGATCGATC |
| LmxM.31.2900 | Distal docking complex protein 2 (dDC2) | Upstream Forward | CTCAATTTCGTTGTTCGACAGGACTATCCTgtataatgcagacctgctgc |
| LmxM.31.2900 | Distal docking complex protein 2 (dDC2) | Upstream Reverse | GTCCTTCTTTTTGGCAGCCACCACTGACATactacccgatcctgatccag |
| LmxM.31.2900 | Distal docking complex protein 2 (dDC2) | 5' sgRNA | GaaattaatacgactcactataggCAAGTCCGCCAATGTACGTCgttttagagctagaaatagc |
| LmxM.31.2900 | Distal docking complex protein 2 (dDC2) | Downstream Forward | AAGAAGAAGGACGCAGGTCAGAAGGCCAAGggttctggtagtggttccgg |
| LmxM.31.2900 | Distal docking complex protein 2 (dDC2) | Downstream Reverse | CTCTCTGCTTTCACCTGGCTGTGACTGCCGccaatttgagagacctgtgc |
| LmxM.31.2900 | Distal docking complex protein 2 (dDC2) | 3' sgRNA | gaaattaatacgactcactataggGTGACCGCATGTCAGCAACAgttttagagctagaaatagc |
| LmxM.31.2900 | Distal docking complex protein 2 (dDC2) | Forward | CTGTGGAAGGTGAGGAGAGGTG |
| LmxM.31.2900 | Distal docking complex protein 2 (dDC2) | Reverse | TCCACCTCCCAGTCTTCTACCA |
| LmxM.24.1030 | Light Chain 1 (LC1) | Upstream Forward | TACGATTTATTGTCCGCACCATCAGTGCCAgtataatgcagacctgctgc |
| LmxM.24.1030 | Light Chain 1 (LC1) | Upstream Reverse | GGCCTCCTTGATGCTCGTCGATGACGACATactacccgatcctgatccag |
| LmxM.24.1030 | Light Chain 1 (LC1) | 5' sgRNA | GaaattaatacgactcactataggGAAGGGAGAGAAGATCAAGGgttttagagctagaaatagc |
| LmxM.24.1030 | Light Chain 1 (LC1) | Downstream Forward | GAGGAAAAAGAGGAGGCGGACCGCCGTCGCggttctggtagtggttccgg |
| LmxM.24.1030 | Light Chain 1 (LC1) | Downstream Reverse | ACACACACACACACACCAACACACACTCACccaatttgagagacctgtgc |
| LmxM.24.1030 | Light Chain 1 (LC1) | 3' sgRNA | gaaattaatacgactcactataggAGAGGTGTGGGGAGCATCTGgttttagagctagaaatagc |
| LmxM.24.1030 | Light Chain 1 (LC1) | Forward | ATGGACAAGGAGATCTCGACGC |
| LmxM.24.1030 | Light Chain 1 (LC1) | Reverse | TCATGTACAGCACCCGAAGTCC |
| LmxM.13.1650 | Outer Arm Dynein β (OADβ) | Upstream Forward | GAAACTGCCTTCTCGCACTGCGCCGGTCAGgtataatgcagacctgctgc |
| LmxM.13.1650 | Outer Arm Dynein β (OADβ) | Upstream Reverse | TGGCGCGTCATCCTTGTCGCCCTTCCCCATactacccgatcctgatccag |
| LmxM.13.1650 | Outer Arm Dynein β (OADβ) | 5' sgRNA | GaaattaatacgactcactataggTTGACCTGAGTGTTTGTCTCgttttagagctagaaatagc |
| LmxM.13.1650 | Outer Arm Dynein β (OADβ) | Downstream Forward | GGGGTGGGTCTGTTGTTAGACGTCGTGGAGggttctggtagtggttccgg |
| LmxM.13.1650 | Outer Arm Dynein β (OADβ) | Downstream Reverse | TGTTTCGTCAGGCACGTGTCGAGCACGCCGccaatttgagagacctgtgc |
| LmxM.13.1650 | Outer Arm Dynein β (OADβ) | 3' sgRNA | gaaattaatacgactcactataggTAACCAGACTCGGACACACGgttttagagctagaaatagc |
| LmxM.13.1650 | Outer Arm Dynein β (OADβ) | Forward | CTGTACTCGGTGAAGTCGCTCA |
| LmxM.13.1650 | Outer Arm Dynein β (OADβ) | Reverse | GTTGTTGTAGAAGCCGGACTGC |
| LmxM.13.0430 | Radial spoke protein 4/6 (RSP4/6) | Upstream Forward | ACGGTCGCGCTCTCTCGAGCCGTCGAATTGgtataatgcagacctgctgc |
| LmxM.13.0430 | Radial spoke protein 4/6 (RSP4/6) | Upstream Reverse | CTCCGCCGCAGAAAGCGCTGTTGAGGCCATactacccgatcctgatccag |
| LmxM.13.0430 | Radial spoke protein 4/6 (RSP4/6) | 5' sgRNA | gaaattaatacgactcactataggAGCTCATTCTCCGCCGACTAgttttagagctagaaatagc |
| LmxM.13.0430 | Radial spoke protein 4/6 (RSP4/6) | Downstream Forward | CCGGAAGAGCTGGAGGAAGAGGAGGACAGCggttctggtagtggttccgg |
| LmxM.13.0430 | Radial spoke protein 4/6 (RSP4/6) | Downstream Reverse | GAGGAAGGGGAAAGGGAGGTTGGATACCCGccaatttgagagacctgtgc |
| LmxM.13.0430 | Radial spoke protein 4/6 (RSP4/6) | 3' sgRNA | gaaattaatacgactcactataggAAAGAGGTTCCGCGGGTGCGgttttagagctagaaatagc |
| LmxM.13.0430 | Radial spoke protein 4/6 (RSP4/6) | Forward | GACTACTTTGTTCTCCGCACGC |
| LmxM.13.0430 | Radial spoke protein 4/6 (RSP4/6) | Reverse | ATGCGGTTACCCAGAACGTGTA |
| LmxM.20.1400 | Paralyzed flagella protein 16 (PF16) | Upstream Forward | CCGCGCTACACAGTGGCCCTCTTCCCTCCTgtataatgcagacctgctgc |
| LmxM.20.1400 | Paralyzed flagella protein 16 (PF16) | Upstream Reverse | GAAGGTTTGCAGAATAACCCGATTCGACATactacccgatcctgatccag |
| LmxM.20.1400 | Paralyzed flagella protein 16 (PF16) | 5' sgRNA | gaaattaatacgactcactataggCTTTGTTTGGCGGACGGGAGgttttagagctagaaatagc |
| LmxM.20.1400 | Paralyzed flagella protein 16 (PF16) | Downstream Forward | AAGATCGAGAACTACCACGTGCAGCAGCACggttctggtagtggttccgg |
| LmxM.20.1400 | Paralyzed flagella protein 16 (PF16) | Downstream Reverse | CGAGCAGCGTGCATGGGCGTGACTGTGCCGccaatttgagagacctgtgc |
| LmxM.20.1400 | Paralyzed flagella protein 16 (PF16) | 3' sgRNA | gaaattaatacgactcactataggCGGATGCTCAGCGGGCCTTTgttttagagctagaaatagc |
| LmxM.20.1400 | Paralyzed flagella protein 16 (PF16) | Forward | CCAGCACTGTGTCTACCTACCC |
| LmxM.20.1400 | Paralyzed flagella protein 16 (PF16) | Reverse | TGATGATCTGGATGGACTCGGC |

**Supplemental Table 2. Primer sequences used for protein tagging, gene deletion and deletion validation.**
