# Supplementary figures and images for: Intraflagellar transport speed is sensitive to genetic and mechanical perturbations to flagellar beating
Source: J Cell Biol. 2024 Jun 3;223(9):e202401154. doi: 10.1083/jcb.202401154 (PMC11148470; doi:10.1083/jcb.202401154)

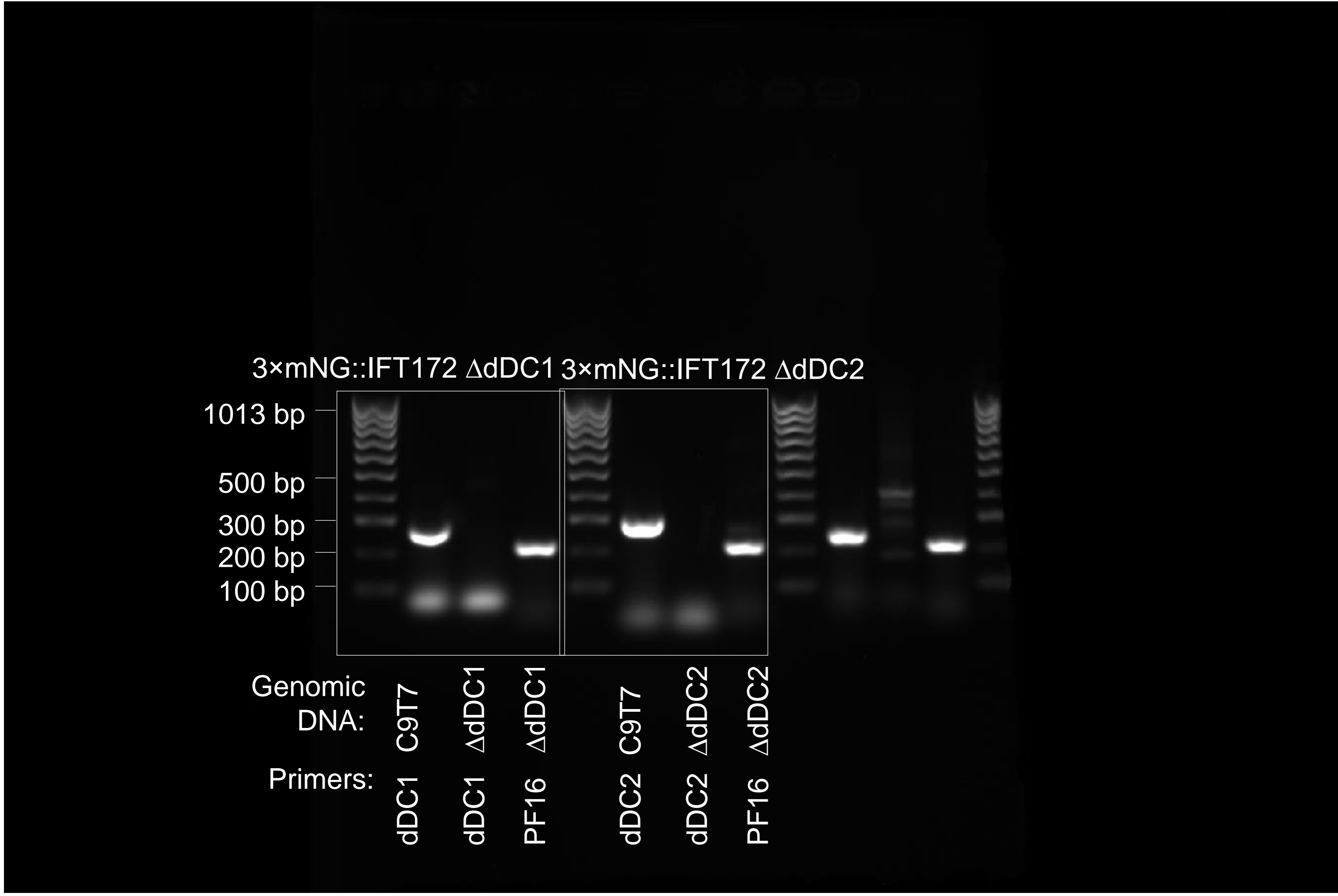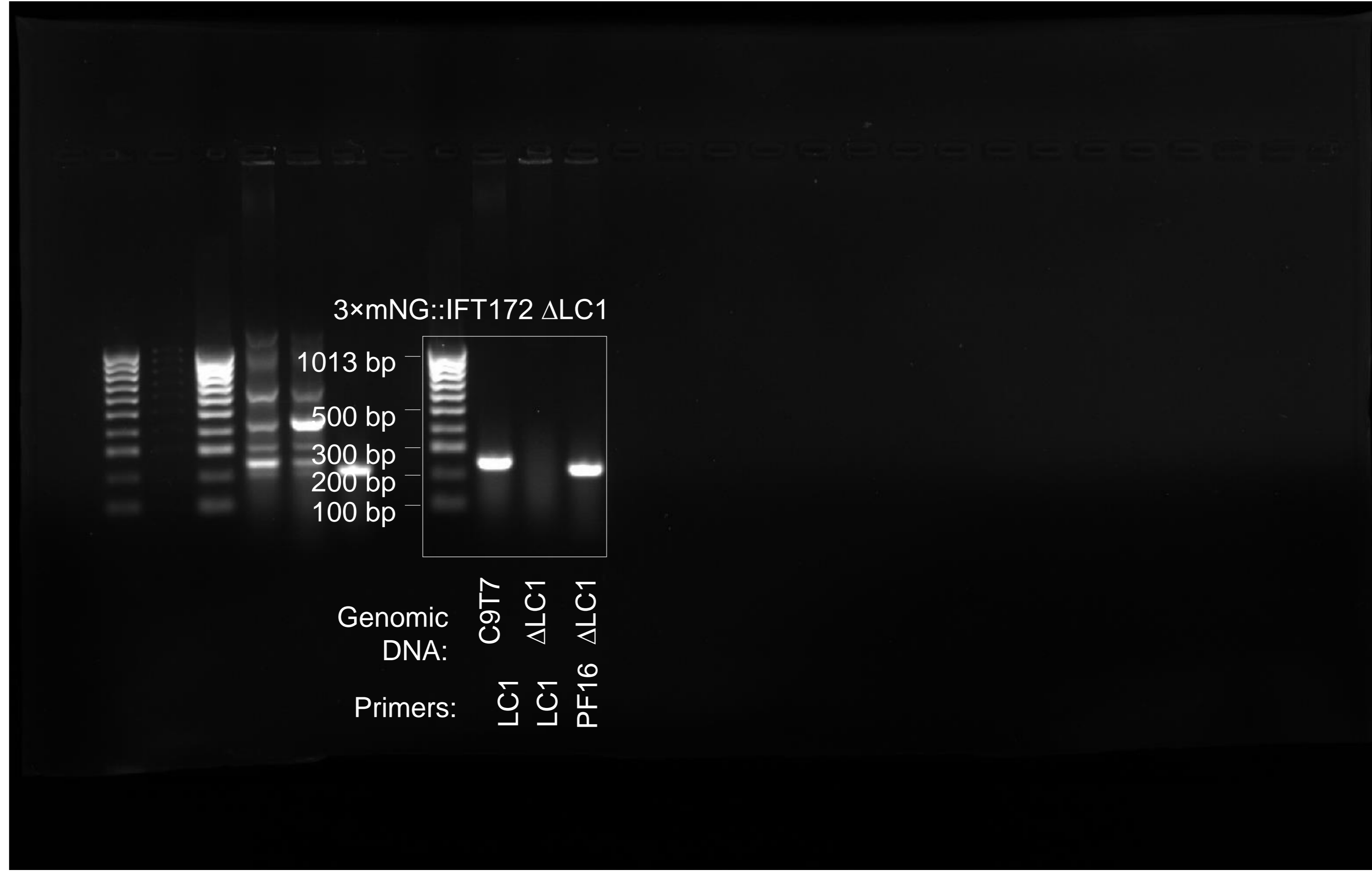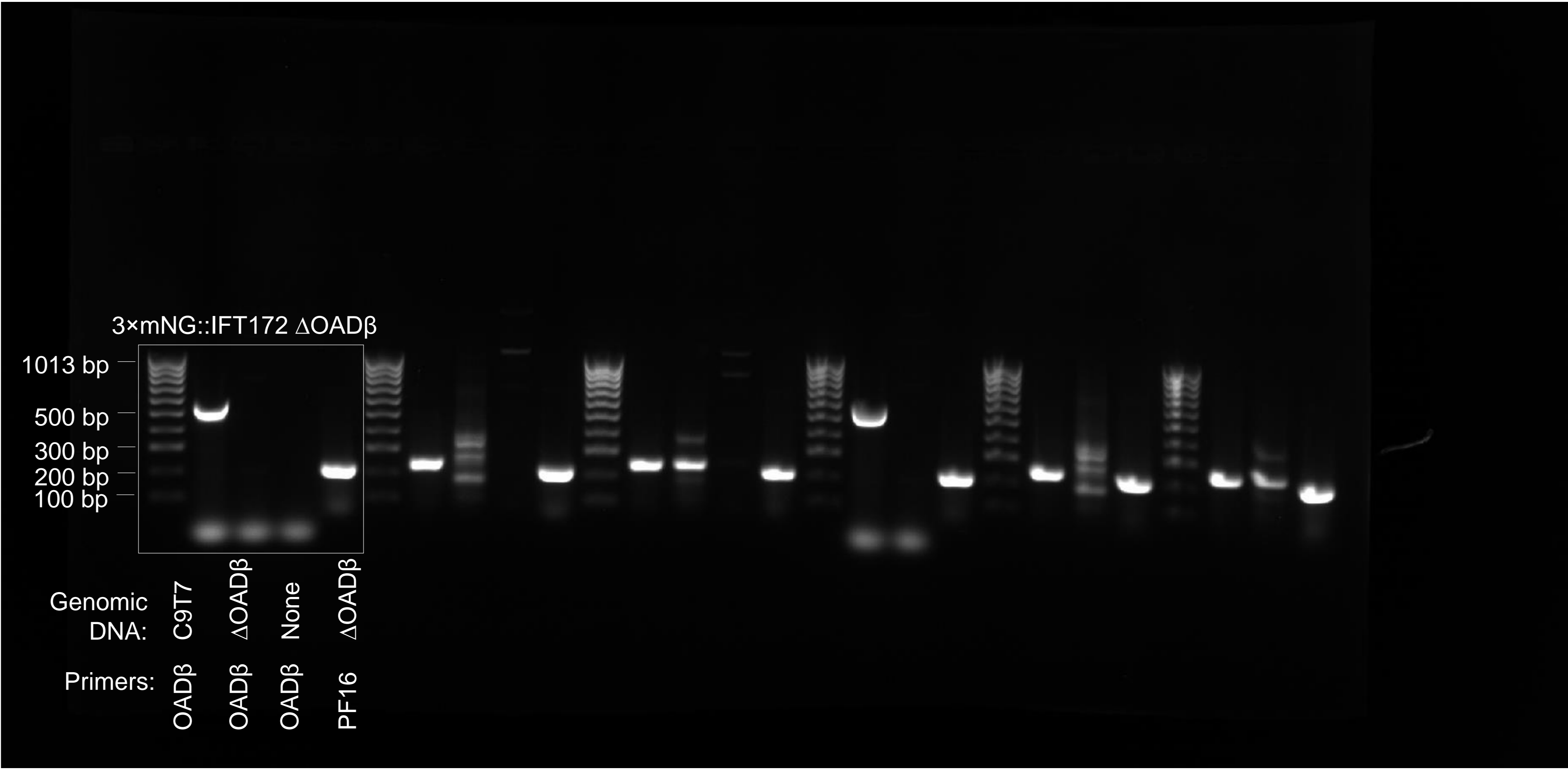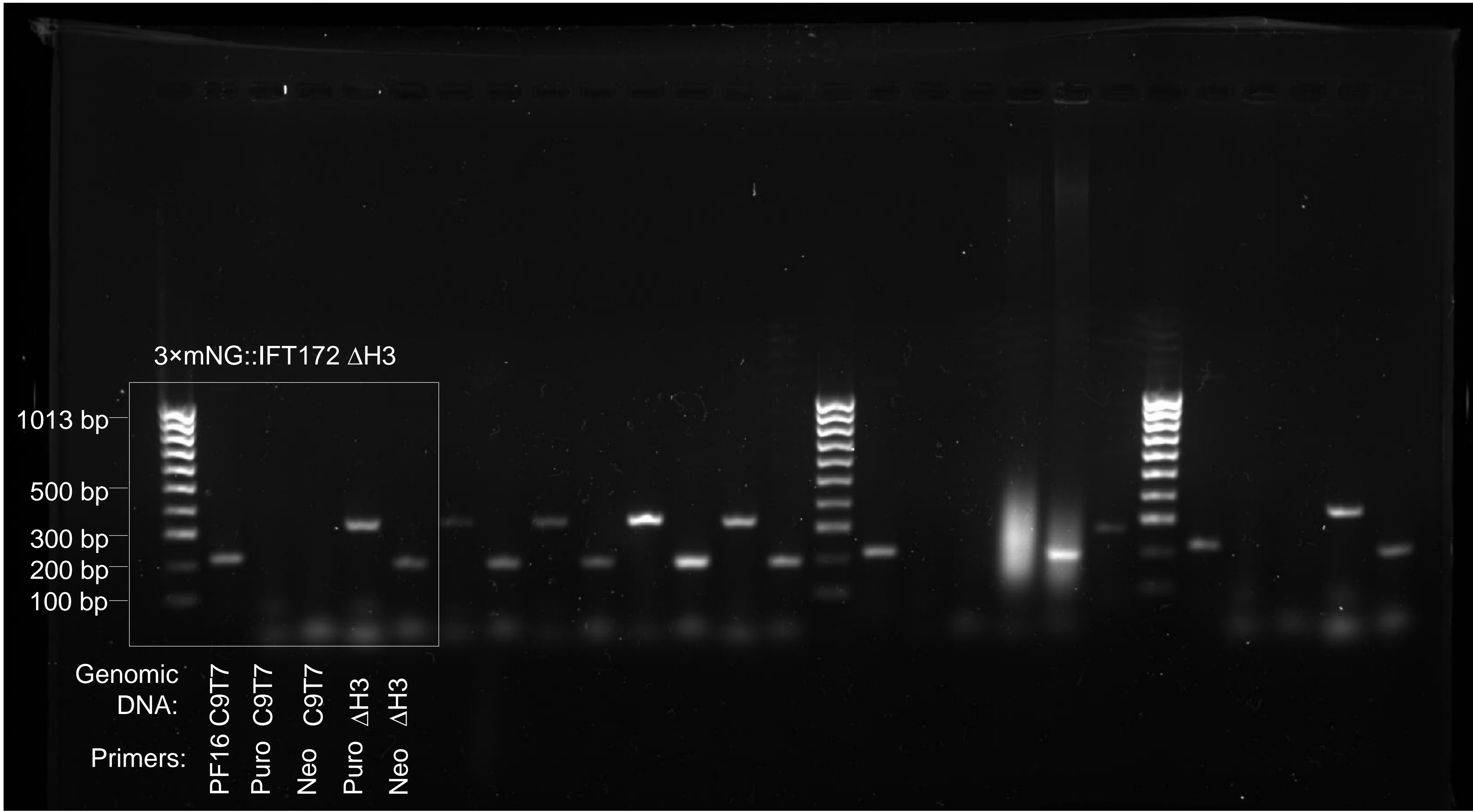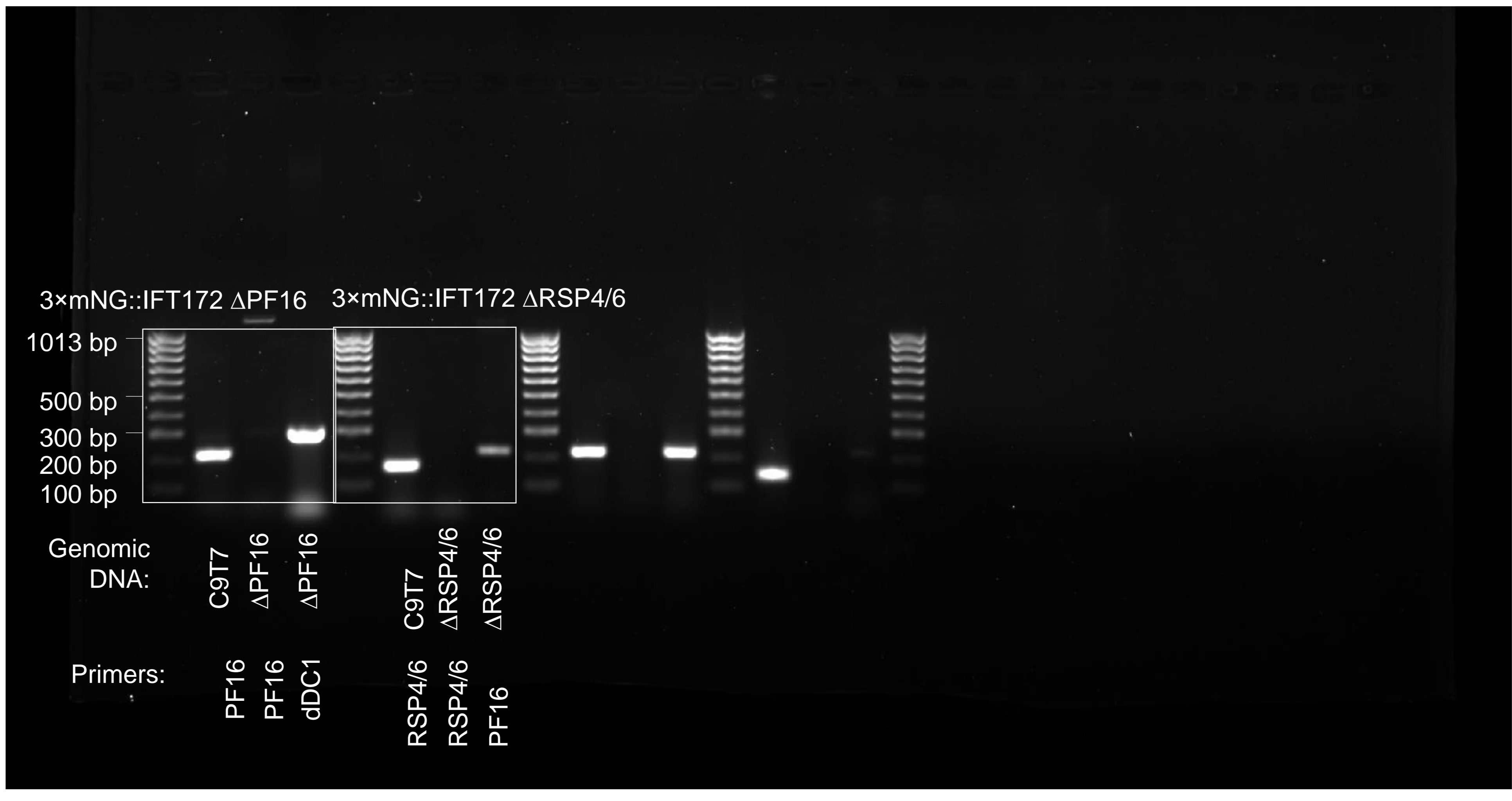

Supplement: SourceData FS3 — is the source file for Fig. S3. [file JCB_202401154_SourceDataFS3.pdf]
